# The combined application of chemical and microbial fertilizers enhanced microbial diversity and improved soil fertility in the peanut rhizosphere within a sugarcane-peanut intercropping system

**DOI:** 10.3389/fmicb.2026.1751211

**Published:** 2026-01-23

**Authors:** Haining Wu, Jiayu Qin, Boyin Li, Zhipeng Huang, Guoting Liao, Xiumei Tang, Zhong Li, Jun Xiong, Zhongkui Gao, Jing Jiang, Ruichun Zhong, Zhuqiang Han, Liangqiong He, Ronghua Tang

**Affiliations:** 1 Cash Crops Research Institute, Guangxi Academy of Agricultural Sciences, Nanning, China; 2 Guangxi Academy of Agricultural Sciences, Nanning, China; 3 Guangxi Crop Genetic Improvement and Biotechnology Laboratory, Guangxi Academy of Agricultural Sciences, Nanning, China

**Keywords:** intercropped peanut, metagenomics, microbial diversity, microbial fertilizers, nitrate reduction genes

## Abstract

The decline in soil microecological balance and fertility caused by continuous cropping obstacles and excessive application of chemical fertilizers has become a critical bottleneck restricting the sustainable development of the peanut industry. However, intercropping can enhance resource utilization efficiency, and microbial fertilizers can improve soil properties and increase nutrient usability. Therefore, we evaluated the effects of six fertilization treatments [no fertilization (CK), 100% chemical fertilizer (T1), microbial fertilizer (T2), 100%chemical fertilizer+microbial fertilizer (T3), 80% chemical fertilizer+microbial fertilizer (T4) and 60% chemical fertilizer+microbial fertilizer (T5)] on chemical properties and microbial communities of the rhizosphere soil of intercropped peanuts. The results showed that compared with T1, the combined application of chemical and microbial fertilizers significantly increased soil organic matter content and alleviated soil acidification. Microbial analysis indicated that the T4 treatment had the highest Shannon diversity, which was significantly higher than T1, demonstrating its effectiveness in reversing the suppressive effect of chemical fertilizer alone on microbial diversity. Principal coordinate analysis and redundancy analysis further confirmed that fertilization significantly altered microbial community structure, with a clear separation between the combined application and chemical-fertilizer-alone treatments, forming a distinct microbial community. Specifically, the T4 treatment significantly increased the abundance of rhizobia. Under T4 treatment, the abundance of assimilatory nitrate reductase genes (such as *nasB* and *NR*) decreased, while that of *narB*, and *nirA* increased; simultaneously, the abundance of dissimilatory nitrate reductase and denitrification-specific genes significantly increased. Mantel test analysis revealed significant positive correlations between soil total nitrogen, available nitrogen content, microbial communities, and crop yield. In summary, the combined application of chemical and microbial fertilizers optimizes the soil microenvironment by synergistically enhancing soil fertility (increasing organic matter, regulating pH) and reshaping microbial community structure (increasing diversity, enriching beneficial bacteria). These findings can provide theoretical basis for the optimization of fertilization strategy in peanut intercropping system.

## 1 Introduction

Peanut is an important oil and economic crop in China, as well as a key cash crop for increasing farmers’ income in dryland regions. It holds a significant position in agricultural production due to its rich nutritional composition (Dai et al., 2018a). However, major peanut-producing areas face challenges such as “soil fertility degradation (e.g., thinning of the plow layer, declining organic matter, nutrient imbalance)” and “water scarcity” (Han et al., 2022; Gao et al., 2009; Yan et al., 2017). Additionally, under traditional monoculture practices, continuous cropping obstacles (e.g., accumulation of rhizosphere pathogens, reduced soil enzyme activity) frequently occur in peanut cultivation, leading to an annual decline in yield (Liu et al., 2020). At the same time, China is confronted with the prominent contradiction of a large population and limited arable land (Wu et al., 2017). Issues such as land resource scarcity and insufficient reserve farmland are becoming increasingly pressing. How to ensure food security and achieve efficient utilization on limited arable land has become a critical challenge for agricultural development.

Under the condition of long-term single cultivation, the toxic effect of accumulated peanut root exudates not only inhibits root growth and nutrient absorption, but also destroys the microecological level of soil (Li et al., 2019; Dai et al., 2019; Xu et al., 2020). This leads to the enrichment of soil-borne disease and a decline in beneficial microbial activity. Furthermore, continuous cropping exacerbates nutrient imbalances (Liu et al., 2015; Bai et al., 2015). Given peanuts’ high demand for phosphorus, potassium, and calcium, prolonged monoculture coupled with unbalanced nutrient supplementation causes a sharp decline in soil available phosphorus and exchangeable calcium, along with the accumulation of unavailable nutrients (Ghosh et al., 2009; Xu et al., 2020). This creates a “demand–supply mismatch” of nutrients and further deteriorates soil productivity. In addition, in order to increase peanut production in the short term, excessive application of chemical fertilizers aggravated the ecological imbalance of soil microorganisms and the decline of fertility (Liu et al., 2022; Hou et al., 2012). And the leaching and volatilization of large amounts of nitrogen lead to soil acidification and water eutrophication, further degrading soil quality (Ju et al., 2009; Guo et al., 2010). Consequently, soil ecological functions are significantly weakened, forming a vicious cycle of “increasing fertilizer use, deteriorating soil quality, and stagnant yield growth” (Zhao et al., 2016).

Intercropping system is an effective method to alleviate continuous cropping obstacles and soil degradation because of its high resource utilization efficiency and eco-friendly characteristics (Yang et al., 2017). For example, peanut-maize intercropping can utilize soil moisture and nutrients more effectively by using root niche complementarity, thus reducing the risk of excessive consumption of specific nutrients; Meanwhile, weeds and pests are reduced (Li et al., 2009; Guo et al., 2021). However, the yield potential of intercropping is constrained by soil health issues (Ghosh et al., 2003). Challenges such as soil acidification and fertility decline can hinder the complementary effects of resource utilization, and may even lead to suboptimal yield increases due to intensified nutrient competition. Soil microbial communities play a crucial role in improving the utilization efficiency of soil nutrients (Nannipieri et al., 2003). Therefore, microbial fertilizers containing soil microorganisms can be used to partially replace chemical fertilizers to improve soil quality. This is because microbial fertilizers can promote the absorption of minerals through the metabolism of beneficial microorganisms they contain (Zhao et al., 2023); induce increased hormone production on root surfaces or reduce the harmful effects of pathogens (Herrmann and Lesueur, 2013); and improve crop yield and quality by enhancing soil physical and biological properties as well as promoting plant nutrient uptake (Yang et al., 2020). Combining intercropping with microbial fertilizers thus achieves soil remediation from both physical and ecological dimensions, significantly improving nutrient use efficiency. This model not only reduces reliance on chemical fertilizers and increases yields but also advances agricultural sustainability and environmental protection. This comprehensive strategy effectively addresses soil degradation while forming a synergistic effect between the crop planting system and soil microorganisms, providing an all-round solution for the sustainable intensification of peanut production systems.

Previous studies have documented the effects of reduced chemical fertilizer application combined with organic amendments on soil quality and microbial diversity (Zhao et al., 2016; Yue et al., 2016; Marschner et al., 2003; Li et al., 2019; Wu et al., 2025). However, field-scale research remains limited regarding the impacts of partial chemical fertilizer substitution with microbial biofertilizers on soil nutrients and microbial communities. This study hypothesized that the synergistic application of reduced chemical fertilizers and microbial biofertilizers influences crop yield through mediating microbial diversity enhancement and soil fertility improvement. To test this hypothesis, a field experiment with six fertilization treatments was conducted in South China. Soil chemical properties were analyzed, and metagenomic sequencing was employed to characterize the soil microbial community structure and function. Redundancy analysis (RDA) and Mantel tests were further applied to examine the relationships among soil properties, microbial communities, and crop yield. This study aims to reveal the response of soil chemical properties and microbial communities to the application of chemical fertilizers combined with microbial fertilizers.

## 2 Materials and methods

### 2.1 Experimental site and design

A field experiment was conducted in 2023 at the Lijian Base Farm of the Guangxi Academy of Agricultural Sciences, located in Nanning (22°50′N, 108°17′E). The region has an average annual temperature of 21.9 °C and receives 1,065 mm of precipitation. The original soil (0–20 cm) before the experiment contained 22.21 g·kg^−1^ organic matter, 0.128% total nitrogen, 0.061% total phosphorus, and 0.312% total potassium, available nitrogen was 86.12 mg kg^−1^, available phosphorus was 60.24 mg kg^−1^, and available potassium was 128.16 mg kg^−1^ and 6.4 of pH.

In this experiment, a randomized block design was adopted. Six treatments included no fertilization as control (CK), 100% chemical fertilizer (T1), microbial fertilizer (T2), 100% chemical fertilizer combined with microbial fertilizer (T3), 80% chemical fertilizer combined with microbial fertilizer (T4) and 60% chemical fertilizer combined with microbial fertilizer (T5). Each treatment was repeated four times, with a total of 24 experimental plots, each with an area of 13 m. Chemical fertilizers were applied at three levels: 100, 80 and 60%, and the corresponding fertilization amounts were 750 kg ha^−1^, 600 kg ha^−1^ and 450 kg ha^−1^, respectively. The application amount of microbial fertilizer is 100 kg ha^−1^. In addition, all treatments (including the control) were uniformly applied with 750 kg ha^−1^ of calcium magnesium phosphate fertilizer as the base fertilizer. Sugarcane and peanut were planted in the sugarcane-peanut intercropping system at a ratio of 1: 3. Among them, the row spacing of peanut and sugarcane is 0.9 m; The hole spacing of sugarcane is 0.6 m and the row spacing is 2.4 m; The peanut row spacing is 0.3 m, and the peanut hole spacing is 12 cm. Apart from fertilization management, all other practices, including irrigation, weed control, and pest management, followed local agricultural protocols.

Fertilizer details are as follows: (1) Compound fertilizer: a granular nitrogen-phosphorus-potassium compound fertilizer (N: P_2_O_5_: K_2_O = 15:15:15) produced by Stanley Agricultural Group Co., Ltd.; (2) Microbial fertilizer: with an organic matter content of 240 g/kg (derived from fermented soybean meal, mushroom residue, plant ash, livestock manure, and straw), co-inoculated with effective microbial agents dominated by Bacillus species such as *Bacillus subtilis* and *Bacillus licheniformis*, and an effective viable count ≥2.5 × 10_9_ CFU/g; (3) Calcium magnesium phosphate fertilizer: produced by Yunnan Kunyang Phosphorus Capital Calcium Magnesium Phosphate Fertilizer Plant, with an available P_2_O_5_ content ≥ 18%.

### 2.2 Peanut yield

At peanut maturity, uproot all peanut plants in each plot for yield measurement. Manually harvest all pods. Thoroughly wash the pods, they were dried in a dryer until the moisture content of peanut pods was about 10%, and the pod weight was measured. Calculate the actual yield (Yield) of the plot using the following equation:


$$\text{Yield}\;(t\;{\mathrm{ha}}^{-1})=M/S$$

Here, M is the weight of peanuts in the plot (t), and S is the area of the plot (ha).

### 2.3 Soil sample collection and analysis

At the peanut maturity stage, five healthy and uniformly growing peanut plants were randomly selected from the experimental plots. The entire plant, along with its rhizosphere soil, was carefully excavated to maintain the original soil structure. The plant was gently shaken to remove loosely adhered soil (bulk soil). Subsequently, a sterile brush was used to carefully collect the soil still tightly adhering to the peanut roots, which was then placed into a sealed bag. The soil collected from the roots of all five plants was combined to form one composite rhizosphere soil sample. Then divided into two subsets: one was air-dried naturally and passed through a 2 mm sieve for chemical analysis, while the other was stored at −80 °C for subsequent microbial analysis.

Soil organic matter was determined using the Walkley-Black method (Walkley and Black, 1934), and total nitrogen (TN) was measured by the Kjeldahl method. Available nitrogen (AN) was determined by the alkali diffusion method. Total phosphorus (TP) was measured using NaOH fusion followed by molybdenum blue colorimetry, and available phosphorus (AP) was extracted and determined by molybdenum-antimony anti-colorimetry. Total potassium (TK) and available potassium (AK) were analyzed by flame photometry. Soil pH was measured in a 1:2.5 (w/v) soil-water suspension using a pH electrode (PHS-3B, China).

### 2.4 Metagenomic analysis

#### 2.4.1 DNA extraction

DNA extraction was performed using the Mag-Bind® Soil DNA Kit (Omega Bio-tek, United States). Following extraction, DNA concentration and purity were measured, and integrity was verified by 1% agarose gel electrophoresis. The DNA was fragmented to approximately 400 bp using a Covaris M220 (Geneco, China) and used for paired-end (PE) library construction. Raw sequencing data have been deposited in the NGDC database (BioProject ID: PRJCA051417).

#### 2.4.2 Metagenomic assembly and gene annotation

Adapter sequences at the 3′ and 5′ ends of reads were trimmed using fastp (Chen et al., 2018). Reads shorter than 50 bp and with an average base quality below 20 after trimming were filtered out to obtain high-quality sequences for subsequent analysis. BWA (Li and Durbin, 2009) was used to align reads to the host DNA sequence, and contaminating reads with high similarity were removed. *De novo* assembly of the processed sequences was performed with MEGAHIT (Li et al., 2015). Contigs with lengths ≥500 bp were selected as the final assembly results. Open reading frames (ORFs) within the assembled contigs were predicted using MetaGene (Noguchi et al., 2006). Genes with nucleotide lengths ≥100 bp were translated into amino acid sequences using Emboss 6.6.0 [9]. A non-redundant gene set was constructed with CD-HIT (Fu et al., 2012). Gene abundance in each sample was quantified using SOAPaligner (Li et al., 2008). Amino acid sequences of the non-redundant gene set were aligned against the NR and KEGG databases using Diamond (Buchfink et al., 2015), and BLASTP (BLAST version 2.2.28+) was applied for taxonomic and functional annotation of each sample.

### 2.5 Data analysis

Prior to conducting the analysis of variance, all data were tested for normal distribution and homogeneity of variance. One-way analysis of variance (ANOVA) was performed to assess the effects of different fertilization treatments on soil chemical properties at the *p* < 0.05 level, with least significant difference (LSD) tests for post-hoc comparisons. Statistical analysis and graphing were conducted using R software (version 3.3.1). The alpha diversity index was calculated using the boot and stats packages in R. Beta diversity was assessed via weighted UniFrac distance and analyzed using PCoA in R. RDA analysis for multiple correlated changes between environmental factors and community composition at the genus level was performed using the vegan package in R. The ReporterScore package in R was employed to identify statistically significant differences in KEGG pathways. Mantel tests using the vegan package in R explored relationships among microbial abundance, soil physicochemical properties, and peanut yield.

## 3 Results

### 3.1 Peanut yield

**Figure 1:**
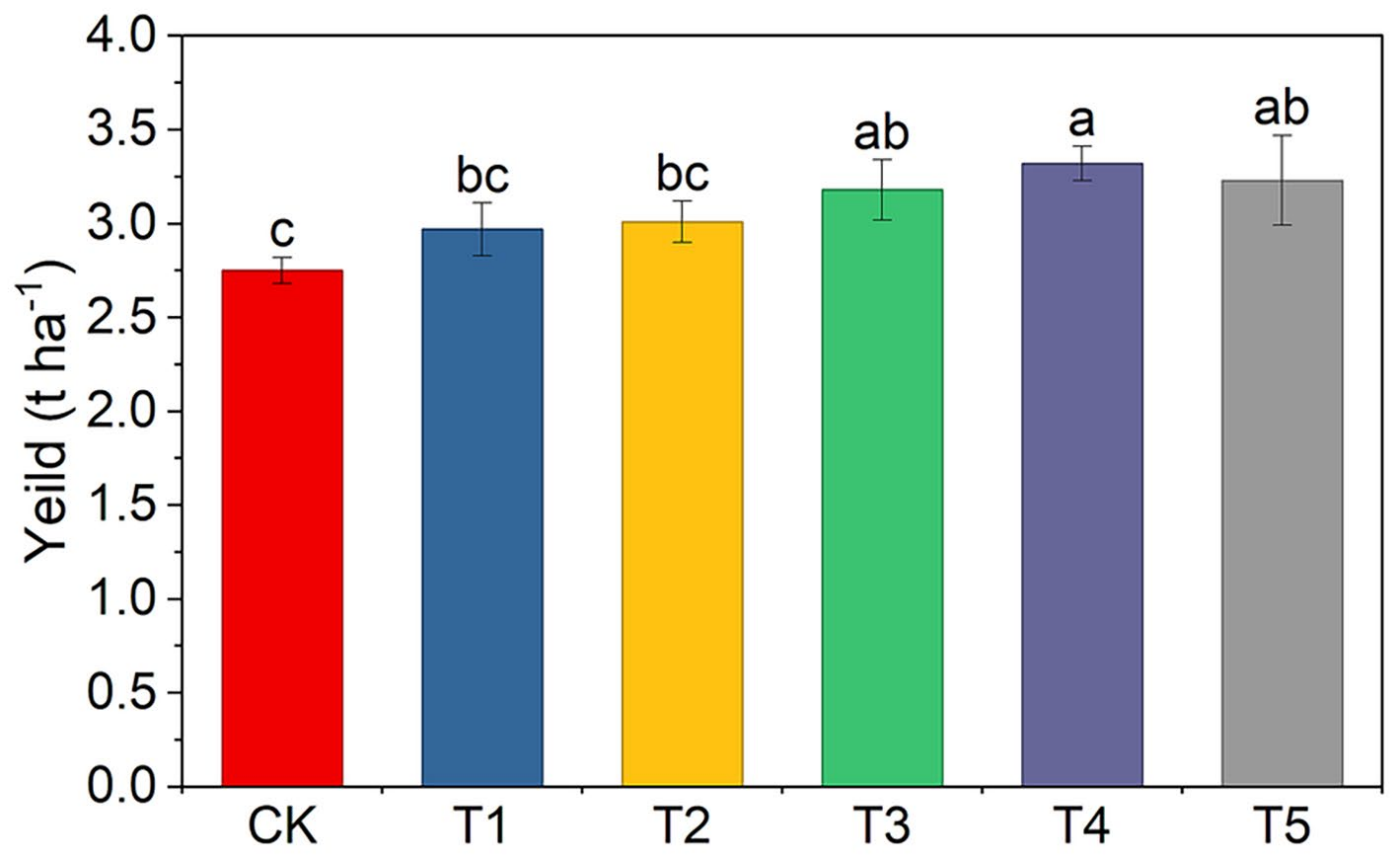
Peanut yield under different fertilization treatments. CK: No fertilization; T1: 100% chemical fertilizer; T2: 100% microbial fertilizer; T3: combined chemical and microbial fertilizer; T4: 80% chemical fertilizer + microbial fertilizer; T5: 60% chemical fertilizer + microbial fertilizer. The data are presented as mean ± standard error (*n* = 4). Different letters indicate significant differences at *p* < 0.05.

Different fertilization treatments had a significant impact on peanut yield. No statistically significant differences were observed among the CK, T1, and T2 treatments. However, compared to CK, the application of chemical fertilizer alone (T1) and microbial fertilizer alone (T2) increased peanut yield by 8.1–9.3%. The combined application of chemical and microbial fertilizers (T3, T4 and T5) further significantly enhanced yield, with an increase of 15.5–20.6% compared to CK. It is worth noting that no significant differences were observed between T1 and T2, T3, or T5. Among all treatments, T4 resulted in the highest peanut yield, showing a significant increase of 11.6% compared to T1 (Figure 1).

### 3.2 Soil chemical properties

Table 1 presents the effects of combined chemical and microbial fertilizer application on soil chemical properties. The results showed that compared with the control (CK), the application of chemical fertilizer alone (T1) significantly decreased soil organic matter content and pH, while significantly increasing the contents of available nitrogen, phosphorus, and potassium. In contrast, the application of microbial fertilizer alone (T2) only led to a significant reduction in available phosphorus content. None of the fertilization treatments had a significant impact on total nitrogen, total phosphorus, or total potassium levels in the soil. Compared to T1, the T4 treatment (80% chemical fertilizer + microbial fertilizer) significantly increased soil organic matter content and pH. The combined application of chemical and microbial fertilizers increased soil organic matter content by 2.6–4.6% compared to chemical fertilizer alone. The combined application of chemical fertilizers and microbial fertilizers can effectively mitigate soil acidification trends. Among all combined fertilization treatments, T4 exhibited the highest levels of organic matter.

**Table 1:** Effects of different fertilization treatments on soil physicochemical properties.

Treatment	SM	TN	AN	TP	AP	TK	AK	pH
CK	30.46 ± 0.57a	1.65 ± 0.08a	66.27 ± 3.98c	1.35 ± 0.04a	95.00 ± 2.91b	0.60 ± 0.11a	224.12 ± 8.01b	6.67 ± 0.02a
T1	29.12 ± 0.33b	1.37 ± 0.10a	116.43 ± 6.91a	1.14 ± 0.03a	113.23 ± 7.73a	0.93 ± 0.06a	256.39 ± 6.30a	6.26 ± 0.05d
T2	30.27 ± 0.33ab	1.55 ± 0.11a	103.37 ± 9.30ab	0.98 ± 0.21a	88.23 ± 3.92b	0.83 ± 0.09a	241.26 ± 6.30ab	6.65 ± 0.02a
T3	29.88 ± 0.57ab	1.50 ± 0.13a	100.80 ± 8.52abc	0.93 ± 0.17a	95.35 ± 0.30a	0.88 ± 0.21a	247.31 ± 3.49ab	6.30 ± 0.03 cd
T4	30.46 ± 0.57a	1.50 ± 0.16a	84.00 ± 28.10abc	1.20 ± 0.40a	108.72 ± 3.13b	0.87 ± 0.14a	250.34 ± 13.64a	6.37 ± 0.03bc
T5	30.07 ± 0.33ab	1.56 ± 0.15a	69.30 ± 4.26bc	1.06 ± 0.10a	91.70 ± 3.76b	0.68 ± 0.08a	242.27 ± 10.91ab	6.40 ± 0.04b

### 3.3 Soil microbial alpha and beta diversity

**Figure 2:**
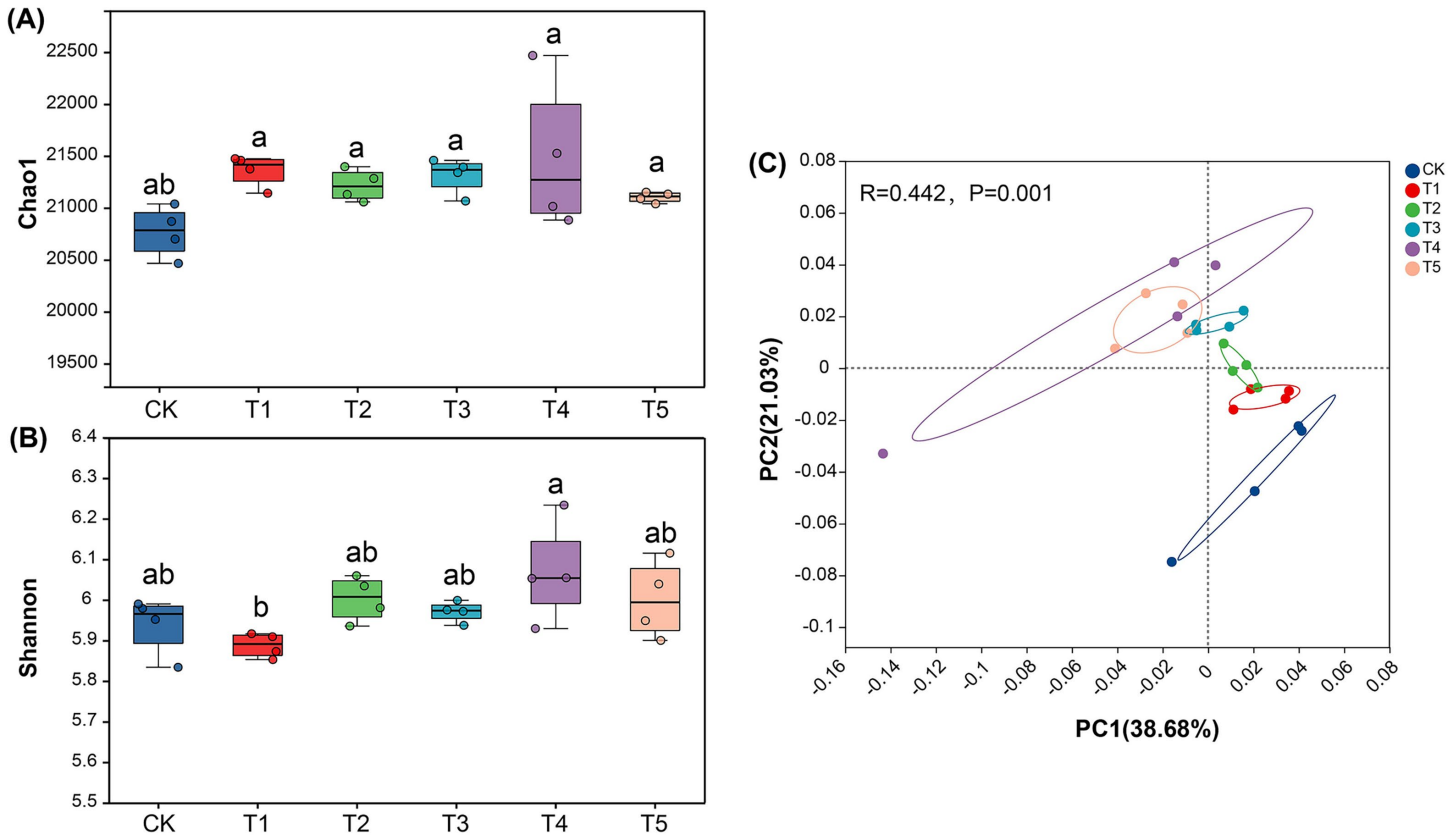
Microbial alpha diversity indices under different fertilization treatments **(A,B)**. Principal coordinate analysis (PCoA) based on weighted UniFrac distance metrics **(C)**. CK: No fertilization; T1: 100% chemical fertilizer; T2: 100% microbial fertilizer; T3: combined chemical and microbial fertilizer; T4: 80% chemical fertilizer + microbial fertilizer; T5: 60% chemical fertilizer + microbial fertilizer. The data are presented as mean ± standard error (*n* = 4). Different letters indicate significant differences at *p* < 0.05.

The Chao1 richness index and Shannon diversity index was used to evaluate bacterial alpha diversity. No significant differences in Chao1 richness were observed among the different fertilization treatments. However, the trend of Chao1 richness followed the order: T4 > T1 > T3 > T2 > T5 > CK (Figure 2A). Among all fertilization treatments, T4 exhibited the highest Shannon diversity, which was significantly greater than that of T1 (Figure 2B). Compared to CK, the application of chemical fertilizer alone reduced microbial Shannon diversity. In contrast, the combined application of chemical and microbial fertilizers enhanced microbial Shannon diversity (Figures 2A,B). Specifically, the integration of microbial fertilizer with chemical fertilizer significantly increased soil microbial Shannon diversity compared to the sole application of chemical fertilizer.

To assess microbial beta diversity, principal coordinate analysis (PCoA) based on weighted Unirac distances was performed on microbial composition. The results demonstrated that fertilization treatments significantly altered the soil microbial community structure compared to CK. Moreover, distinct separation was observed between combined chemical-microbial fertilizer treatments and chemical-fertilizer-only applications (Figure 2C). The close clustering of T1 and T2 (sole application treatments) indicated their similar impacts on microbial communities, likely establishing specific microbial taxa associated with chemical fertilizer application. In contrast, T3 and T4 (combined fertilizer treatments) formed a separate cluster, demonstrating that integrated chemical-microbial fertilization shaped a unique microbial community structure distinct from chemical fertilizer alone. This confirms that microbial fertilizer addition serves not only as nutrient supplementation but also as an effective regulator of microecological dynamics.

### 3.4 Soil microbial taxonomic and functional composition

**Figure 3:**
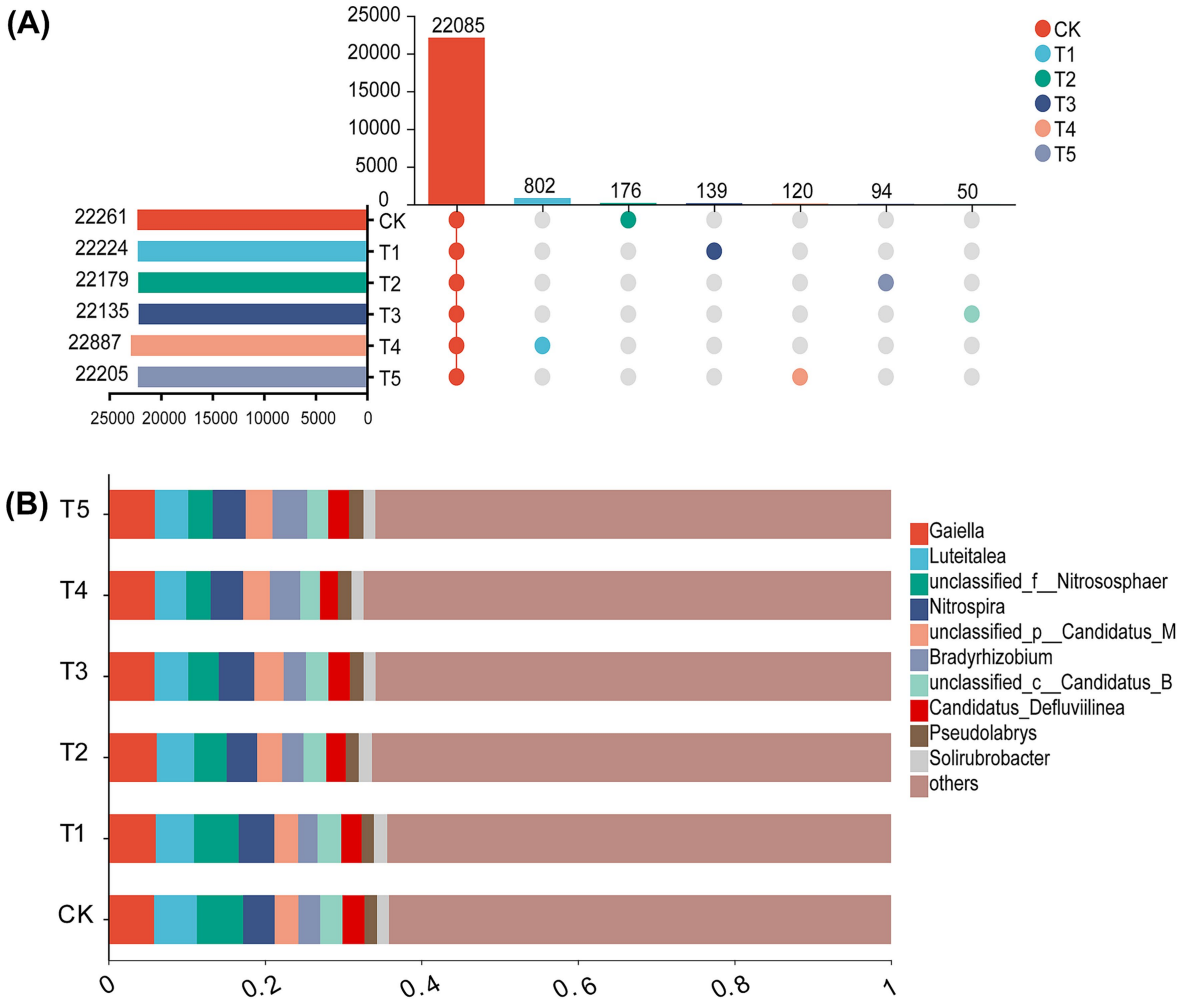
Soil microbial species number **(A)** and microbial community composition at the phylum level **(B)** under different fertilization treatments: CK, no fertilization; T1, 100% chemical fertilizer; T2, 100% microbial fertilizer; T3, combined chemical and microbial fertilizer; T4, 80% chemical fertilizer + microbial fertilizer; T5, 60% chemical fertilizer + microbial fertilizer.

Compared to CK, fertilization treatments significantly altered soil microbial composition. A total of 22,085 microbial species were shared across all treatments. The T4 treatment exhibited the highest number of unique species (802). The trend in unique species richness followed the order: T4 > CK > T1 > T5 > T3 > T2 (Figure 3A).

The phyla Actinomyceta, Pseudomonadota, Acidobacteriota, and Chloroflexota collectively accounted for over 60% of the total sequences (Figure 3B). Significant differences were observed in the relative abundances of Pseudomonodota, Nitrososphaerota, Nitrospirota, and Candidatus_Methylomirabilota across different fertilization treatments. Compared to the CK group, the application of chemical fertilizer alone significantly reduced the relative abundance of Pseudomonodota while increasing the relative abundances of Nitrososphaerota and Nitrospirota. In contrast, the application of microbial fertilizer alone increased the relative abundance of Pseudomonodota and decreased the relative abundances of Nitrososphaerota and Nitrospirota. Among all treatments, the T4 group exhibited the highest relative abundance of Pseudomonodota and the lowest relative abundance of Nitrososphaerota, while the T1 group showed the highest relative abundance of Nitrososphaerota and the lowest relative abundance of Pseudomonodota.

**Figure 4:**
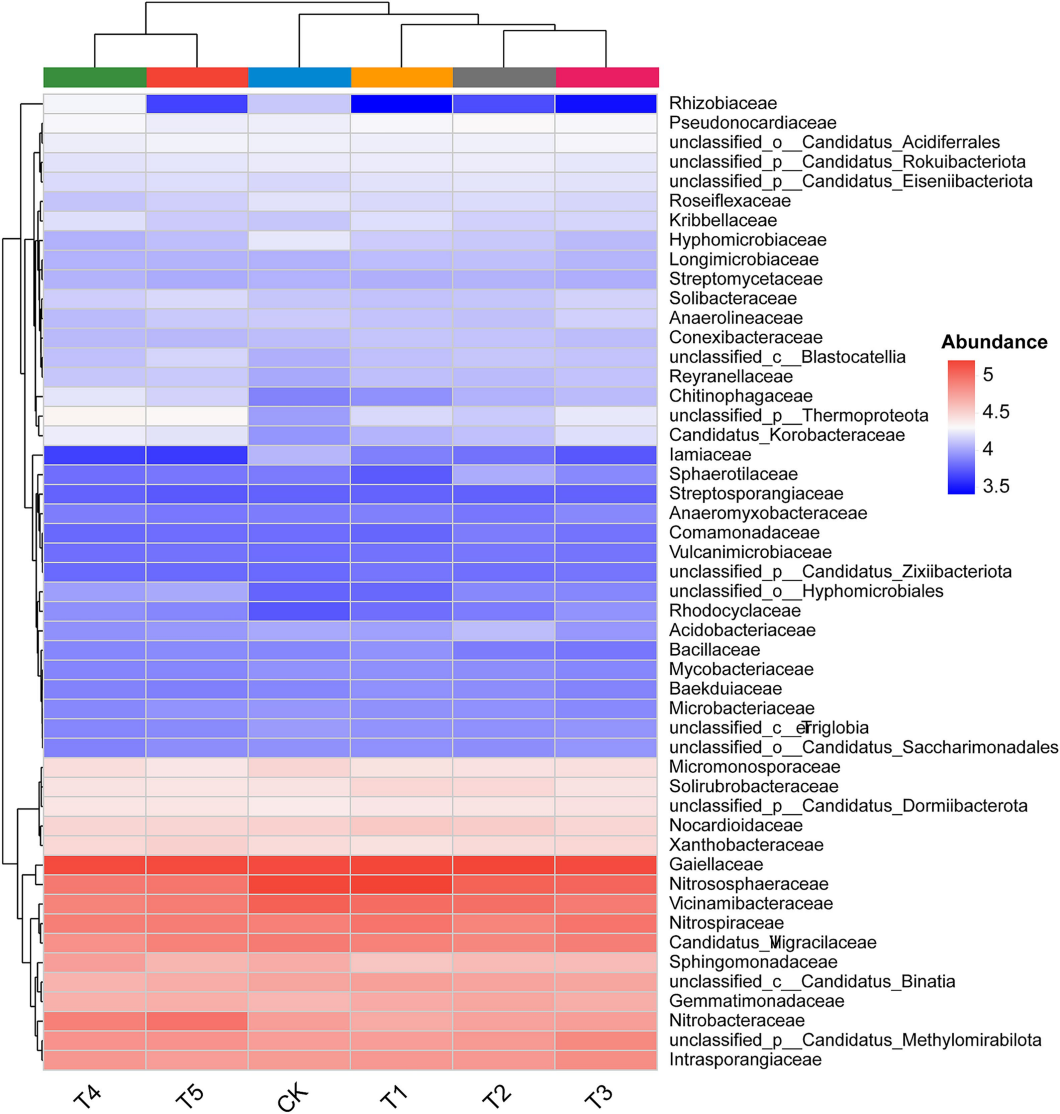
Heatmaps showing the relative abundance of soil microbial OTUs on family levels under different fertilization treatments. CK: No fertilization; T1: 100% chemical fertilizer; T2: 100% microbial fertilizer; T3: combined chemical and microbial fertilizer; T4: 80% chemical fertilizer + microbial fertilizer; T5: 60% chemical fertilizer + microbial fertilizer.

To visualize differences in the relative abundance of microbial communities, a heatmap was constructed at the family level (Figure 4). The heatmap revealed distinct clustering patterns according to different fertilization treatments. Upon closer examination, clear separations were observed among T4, T1, and CK. The standardized value for Rhizobiaceae was 4.27 under the T4 treatment, while it measured 4.15 in T1 and 3.52 in CK, respectively.

Three-level KEGG pathway enrichment analysis was conducted on the control group (CK), single chemical fertilizer application group (T1), single microbial fertilizer application group (T2), and combined chemical and microbial fertilizer application group (T4). This analysis revealed persistent differences in KEGG pathway patterns among different fertilization treatments.

**Figure 5:**
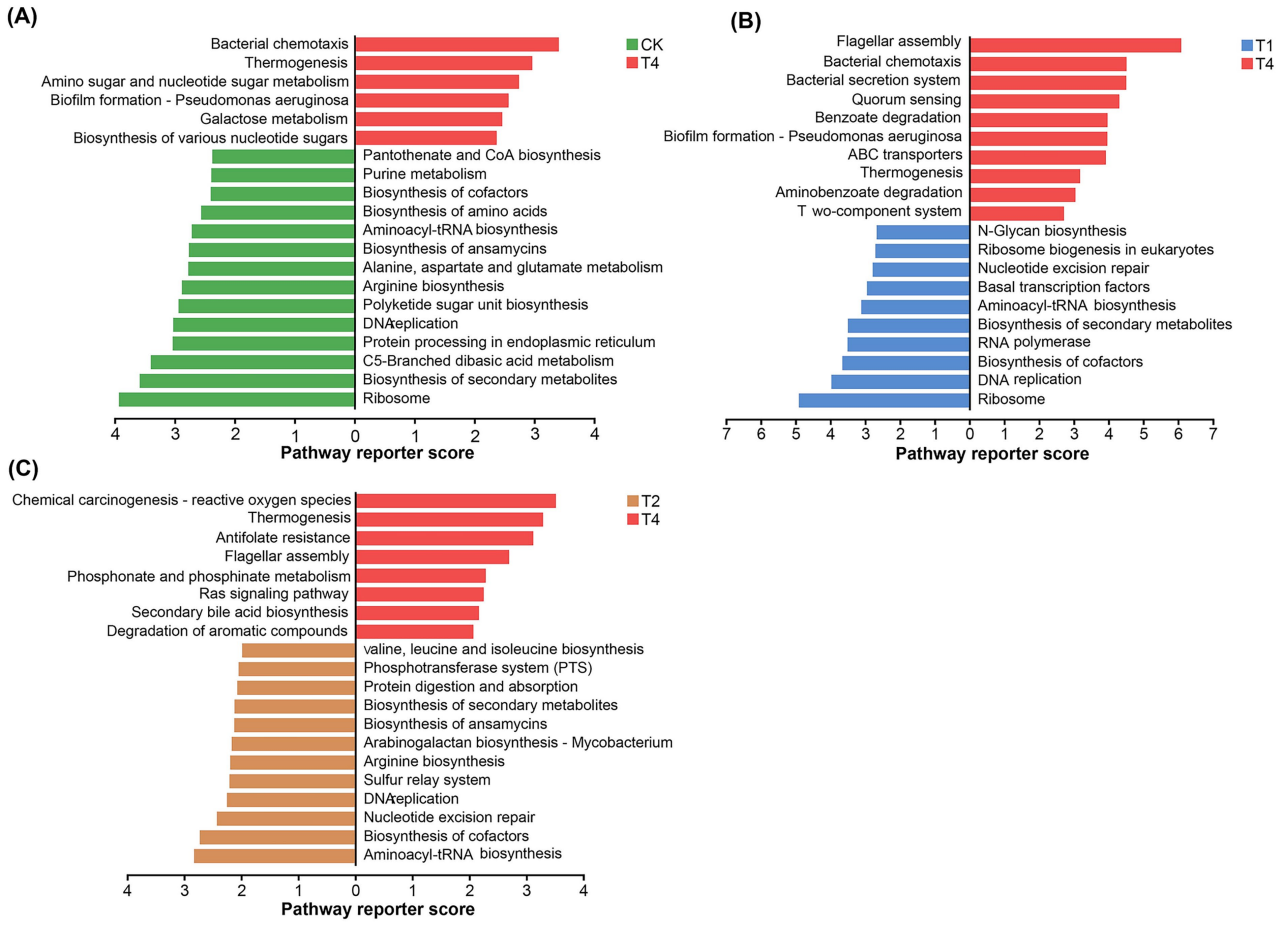
Enrichment analysis of level 3 KEGG pathways based on significant differences among different fertilization treatments: **(A)** CK vs. T4; **(B)** T1 vs. T4; **(C)** T2 vs. T4.

When comparing CK and T4, the pathways primarily associated with T4 included: Bacterial chemotaxis, Thermogenesis, Amino sugar and nucleotide sugar metabolism, Biofilm formation - *Pseudomonas aeruginosa*, Biosynthesis of various nucleotide sugars, and Galactose metabolism (Figure 5A).

When comparing T1 and T4, the pathways mainly linked to T4 were: Flagellar assembly, Bacterial chemotaxis, Bacterial secretion system, Quorum sensing, Benzoate degradation, Biofilm formation - *Pseudomonas aeruginosa*, ABC transporters, Thermogenesis, Aminobenzoate degradation, as well as T wo-component system (Figure 5B).

When comparing T2 and T4, the pathways predominantly correlated with T4 comprised: Chemical carcinogenesis—reactive oxygen species, Thermogenesis, Antifolate resistance, Flagellar assembly, Phosphonate and phosphinate metabolism, Ras signaling pathway, Secondary bile acid biosynthesis, and Degradation of aromatic compounds (Figure 5C).

**Figure 6:**
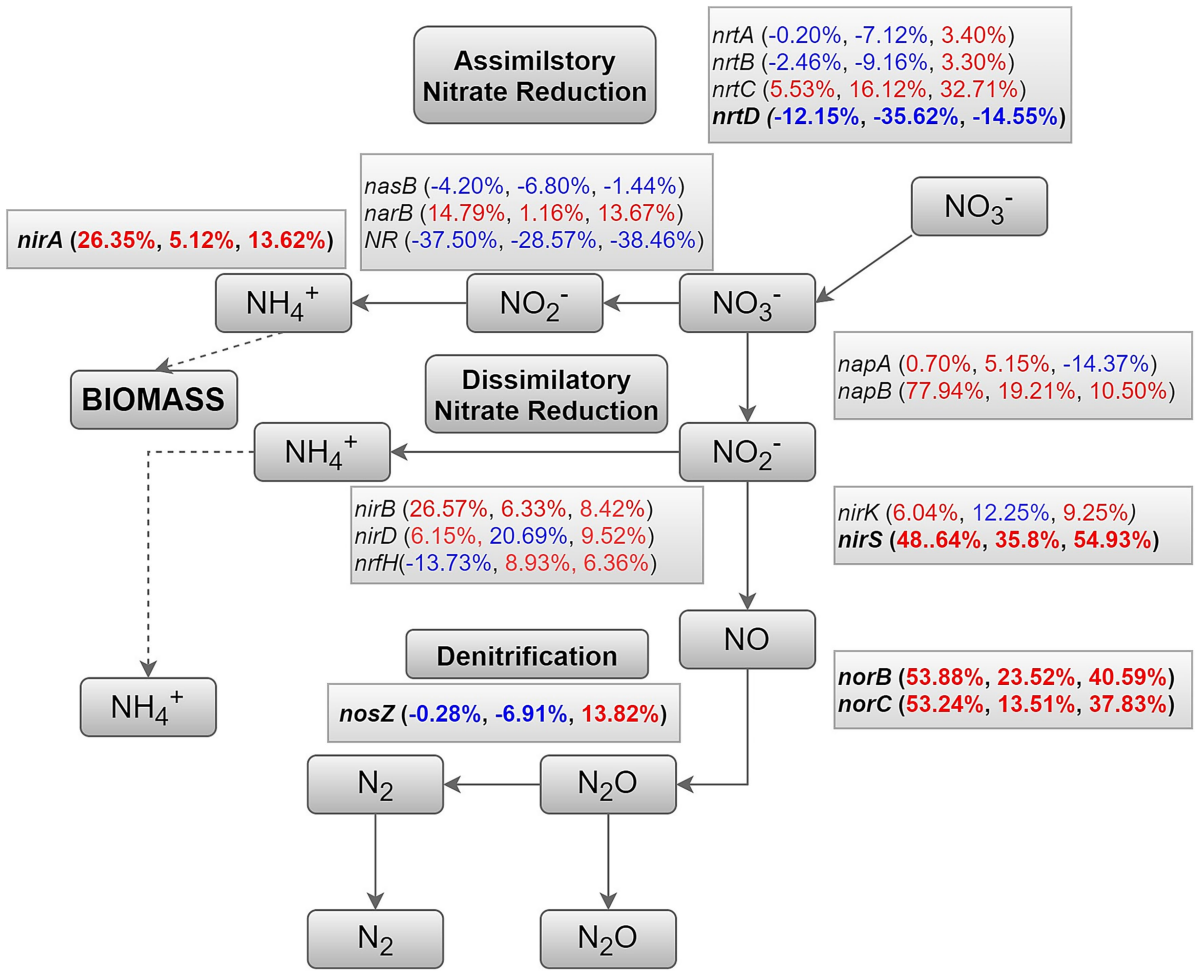
A diagram representing selected nitrogen pathways. Percentages are ordered like this: T4 vs. CK; T4 vs. T1; and T4 vs. T2. Red or green percentages represent an increase or decrease in the abundance of the corresponding genes, respectively.

The results showed that various enzymes encoded by corresponding functional genes are involved in the nitrogen metabolism of rhizosphere soil. However, the abundances of genes related to nitrogen cycling exhibited various changes (Figure 6). Compared with CK, T1 and T2, the abundances of assimilatory nitrate reductase genes *nasB* and *NR* decreased in T4, while those of *narB* increased. In addition, genes specific to dissimilatory nitrate reduction, including napA and *napB*, showed increased abundances after the combined application of chemical fertilizer and microbial fertilizer. Genes specific to denitrification, such as *nirK/S*, *norBC*, and *nosZ*, were significantly higher in the T4 treatment than in CK, T1 and T2. However, genes related to extracellular nitrate transport, including *nrtABD* except *nrtC*, were more abundant in CK and T1 groups than in the T4 group. Particularly, the abundance of the *nrtD* gene in T4 decreased by more than 12.15 and 35.62% compared with CK and T1, respectively.

### 3.5 Relationships between soil microbial communities and environmental factors

**Figure 7:**
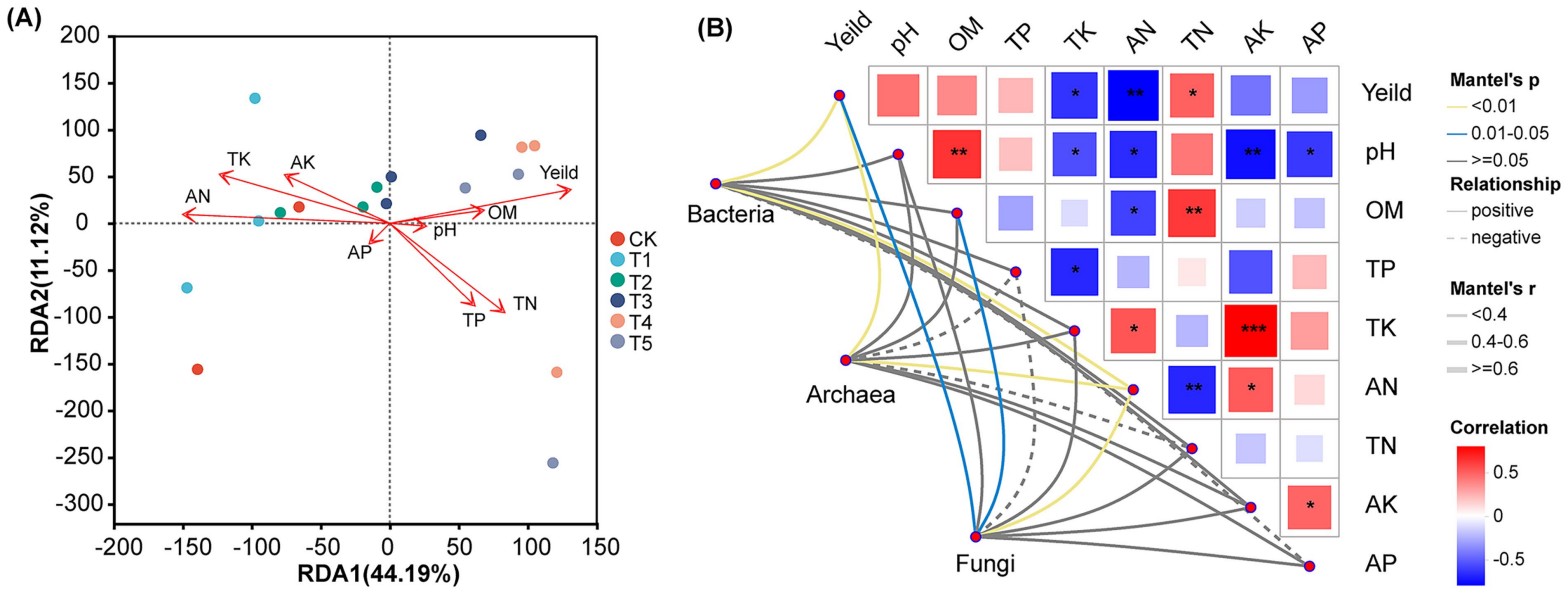
Redundancy analysis of microbial communities, peanut yield, and soil chemical properties **(A)** and pairwise comparisons of soil variables **(B)**.

Multiple comparisons in redundancy analysis (RDA) revealed significant separation in microbial community composition among different fertilization treatments. The results indicated that the first two RDA axes explained 44.19 and 11.12% of the total variance in microbial communities under different fertilization regimes, respectively (Figure 7A). All fertilization treatments (T1-T5) were significantly separated from the control (CK) along the RDA1 axis, demonstrating that fertilization is the primary factor altering the soil microbial community structure. Furthermore, organic matter, pH, and total nitrogen were closely associated with the microbial community distribution in T4 and T5. In contrast, total potassium, available nitrogen, and available potassium were correlated with the communities in T1 and T2.

Mantel test results revealed significant correlations between peanut yield and total nitrogen, total potassium, as well as available nitrogen. Furthermore, significant associations were observed among fungi, actinobacteria, bacterial communities, and both peanut yield and available nitrogen content (Figure 7B).

## 4 Discussion

### 4.1 Effects of combined chemical and microbial fertilizer application on soil chemical properties in intercropped peanuts

Soil chemical properties serve as effective indicators for assessing soil fertility and crop nutrient uptake, and are significantly influenced by farmland fertilization management. This study compared the differences in soil organic matter, pH, and nitrogen, phosphorus, and potassium contents in intercropped peanut systems under different fertilization treatments. Our findings clearly reveal the “dual nature” of chemical fertilizer application alone: compared to CK, it significantly reduced soil organic matter content and pH while markedly increasing available nitrogen, phosphorus, and potassium contents (Table 1). This is because chemical fertilizers, particularly ammonium-based nitrogen fertilizers, release H^+^ ions during soil nitrification, directly leading to decreased soil pH (Bohoussou et al., 2025). Soil acidification accelerates the leaching of base cations such as calcium and magnesium, further deteriorating soil structure. Meanwhile, the rapid availability of chemical fertilizers causes a sharp short-term increase in soil microbial activity, triggering a “priming effect” that accelerates the decomposition of native soil organic matter for energy, resulting in the depletion of organic matter (2024). The reduction in organic matter not only weakens the soil’s capacity to retain nutrients and water but also undermines the foundation for forming stable aggregates (Rabot et al., 2018). However, the input of chemical fertilizers directly introduces high concentrations of mineral nutrients into the soil, significantly enhancing the available nitrogen, phosphorus, and potassium pools in the short term. This explains why chemical fertilizer alone often achieves yield increases in the current season. Nevertheless, this nutrient enrichment comes at the cost of long-term soil health.

In our research results, “only applying microbial fertilizer can significantly reduce the available phosphorus” (Table 1). This shows that the function of microbial fertilizer will be limited if there is no sufficient “substrate” provided by chemical fertilizer. The likely mechanism for the reduction in available phosphorus is that microorganisms require fixed carbon, nitrogen, and phosphorus for cellular construction during their life activities. When external nutrient inputs are insufficient, they may immobilize a portion of the available phosphorus in the soil solution, temporarily reducing its plant availability (Ding et al., 2024). However, it has no significant effects on organic matter, pH, and other macronutrients suggests that microbial fertilizers do not directly supply large quantities of nutrients (Table 1). Their core function lies in “regulation” rather than “supply,” and their potential for soil improvement remains underutilized when applied alone. In contrast, the combined application successfully balances nutrient enhancement with soil health improvement. “Compared to chemical fertilizer alone, the combined application of chemical and microbial fertilizers increased soil organic matter content by 2.6–4.6% while effectively mitigating soil acidification (Table 1).” This is consistent with the research results of Han et al. (2021). This synergistic effect primarily stems from the following processes: First, functional strains in microbial fertilizers promote crop root development through their metabolic activities, increasing rhizodeposits and root biomass (Zhao et al., 2022). Simultaneously, microbial metabolites such as polysaccharides and lipids act as organic binding agents, facilitating the formation of soil aggregates and physically protecting organic carbon from decomposition (Zhang et al., 2022a). The microbial fertilizer used in this study is formulated with functional strains of the Bacillus genus as its core components. The characteristics of these two widely applied plant growth-promoting rhizobacteria provide a species-level mechanism for explaining the synergistic effects observed. Firstly, *Bacillus subtilis* is renowned for its robust secretion system, capable of producing abundant extracellular enzymes such as proteases and cellulases, as well as antimicrobial substances. These metabolites not only degrade difficult-to-decompose organic residues in the soil, promoting organic matter cycling, but the lipopeptides produced also serve as high-quality organic binding agents, directly facilitating the formation of soil aggregates. This may be one of the microscopic mechanisms underlying the significant increase in soil organic matter content in the combined treatment (particularly T4). Secondly, *Bacillus licheniformis* exhibits outstanding capabilities in solubilizing phosphorus and potassium. It secretes organic acids to dissolve fixed inorganic phosphorus and potassium in the soil, converting them into available forms. This function complements the readily available nutrients supplied by chemical fertilizers, creating a “rapid-release + slow-release” complementary supply pattern. This not only explains why available phosphorus and potassium remained at relatively high levels under the T4 treatment but also alleviates the nutrient loss pressure caused by the sole application of chemical fertilizers. Therefore, the effect of the combined fertilization is not merely a simple additive outcome of “chemical” and “biological” inputs; rather, it represents a targeted coupling between the biological properties of specific functional strains and the chemical nature of fertilizers within the soil micro-environment.

Additionally, microbial biomass itself serves as an important source of soil organic matter. Second, functional microorganisms enhance crop uptake efficiency of ammonium and nitrate nitrogen, reducing residual soil nitrogen available for nitrification and thereby decreasing H^+^ production at the source (Ingraffia et al., 2019). Moreover, healthy root systems and abundant organic matter enhance soil buffering capacity, increasing soil resistance to pH changes. Third, chemical fertilizers provide ample energy (e.g., nitrogen) for microorganisms, while the microorganisms significantly improve chemical fertilizer utilization efficiency through biological nitrogen fixation, solubilization of fixed soil phosphorus and potassium, and secretion of phytohormones that promote root absorption (Liu et al., 2025). This establishes a virtuous cycle of “chemical fertilizers feeding microorganisms, and microorganisms promoting peanuts.” This explains why, among all combined treatments, “T4 exhibited the highest levels of organic matter, total nitrogen, total phosphorus, and available potassium.” The T4 treatment likely represents the optimal ratio under the experimental conditions, where the specific microbial inoculant and chemical fertilizer type and dosage achieved the best match, maximizing microbial functionality and resulting in superior performance in enhancing key soil chemical indicators. Although the study results indicate that “fertilization treatments had no significant impact on total nitrogen, total phosphorus, or total potassium contents in the soil,” T4 significantly increased these total nutrients, suggesting that microorganisms may promote the cycling and retention of nutrients within the soil–plant system. In summary, single fertilization strategies have significant limitations in their impact on soil chemical properties in intercropped peanut systems, whereas the combined application of chemical and microbial fertilizers exhibits synergistic advantages in ensuring nutrient supply and improving soil health, representing an effective fertilization model for sustainable agriculture.

### 4.2 Effects of combined chemical and microbial fertilizer application on microbial diversity

This study provides an in-depth investigation into the regulatory effects of different fertilization regimes on soil microbial ecology. Our results demonstrate that the application of chemical fertilizer alone (T1) significantly reduced the Shannon diversity index of soil microorganisms (Figure 2B), which is consistent with previous research findings (Zhang et al., 2022b). Long-term single application of chemical fertilizers leads to soil acidification, salinization, and nutrient imbalance, which in turn inhibits microbial activity. This particularly affects environmentally sensitive functional microbial communities, resulting in a decline in microbial diversity (Dai et al., 2018b). However, when chemical fertilizers were applied in combination with microbial fertilizers, Shannon diversity was increased, with the T4 treatment reaching the highest level (Figure 2B). This strongly demonstrates that the introduction of microbial fertilizers not only neutralizes or mitigates the negative effects of chemical fertilizers, but also creates a more suitable and diverse microenvironment (Tao et al., 2020). This is achieved through the exogenous beneficial microorganisms they carry, the “priming effect” they provide to indigenous microorganisms, and the additional carbon sources they supply, collectively promoting the growth of soil microbial communities. Although no significant differences were observed in Chao1 richness among treatments, the T4 treatment consistently exhibited the highest Chao1 richness (Figure 2A), indicating that the combined application treatment positively influenced both total species abundance and community evenness. Furthermore, PCoA analysis indicated that fertilization treatments significantly altered microbial community structure (Figure 2C), a conclusion further validated and supplemented by RDA analysis results (Figure 7A). Especially, as the combined application treatments (T3, T4, T5) formed distinct clusters compared to the sole application of chemical fertilizer (T1). This indicates that the combined application of chemical fertilizer with microbial fertilizer resulted in a more complex and functionally balanced community pattern. Tang et al. (2023) also reached similar conclusions: the co-application of chemical fertilizers with organic fertilizers (containing abundant functional microorganisms) can enhance the complexity and diversity of bacterial and fungal networks while altering microbial community composition. This alteration in community structure was specifically reflected at the phylum and family levels. For instance, the Pseudomonas phylum exhibited the highest relative abundance in the T4 treatment, while the Acidobacteria phylum showed the lowest abundance (Figure 3). This finding was the exact opposite of the results observed in the T1 treatment. This finding was consistent with previous study indicating (Tao et al., 2020) that the addition of organic matter or microbial fertilizers enriches beneficial bacterial phyla such as Pseudomonas, thereby optimizing the rhizosphere microenvironment. The higher standardized values for families such as Rhizobiaceae in the T4 treatment (Figure 4) further confirm, at the family level, the successful colonization and enrichment of beneficial microbial groups. It is noteworthy that the reshaping of the microbial community structure is closely related to the inoculated functional strains. Although high-throughput sequencing may struggle to fully distinguish exogenously added Bacillus strains from highly homologous indigenous strains at the genus level, the observed changes in the relative abundance of Bacillaceae-related taxa in the combined fertilization treatments (T3, T4, T5), as well as the significant enrichment of gene pathways associated with nutrient cycling and biofilm formation in the T4 treatment (Figure 5), indirectly confirm the successful colonization and active metabolism of the exogenous functional strains. Therefore, the effectiveness of microbial fertilizers depends not only on their “presence” but, more importantly, on the composition of their core bacterial species. The optimized community structure and functions observed in this study represent a steady-state outcome resulting from complex interactions between the indigenous microbiome and inoculants dominated by Bacillus strains with broad-spectrum growth-promoting capabilities, driven by the energy supplied by chemical fertilizers.

Through KEGG pathway enrichment analysis, we found that the T4 treatment significantly enriched multiple functional pathways closely related to microbial life activities. Compared to CK, T1, or T2, pathways such as “quorum sensing,” “flagellar assembly,” “bacterial chemotaxis,” and “biofilm formation in *Pseudomonas aeruginosa*” showed sustained high expression in the T4 treatment (Figure 5). This strongly suggests that the microbial community exhibits enhanced motility, colonization, and biofilm formation capabilities. These abilities are prerequisites for microorganisms to successfully establish themselves and function within the rhizosphere (Vannier et al., 2023). Additionally, the enrichment of metabolic pathways such as “benzoate degradation” indicates that the T4 treatment group exhibits enhanced carbon utilization capacity. This is consistent with the results of an experiment combining chemical fertilizer application with biogas slurry (Yang W. et al., 2024; Yang Y. et al., 2024). At the functional gene level of the nitrogen cycle, T4 treatment demonstrated a more efficient and greener nitrogen transformation pattern. In our findings, the abundance of genes responsible for nitrate assimilation into ammonium, such as *napB*, increased. Meanwhile, key denitrification genes including *nirK/S*, *norBC*, and *nosZ* were significantly upregulated under T4 treatment. Notably, the abundance of the *nosZ* gene (nitrous oxide reductase) showed a marked increase (Figure 6). This is consistent with the results of an experiment on fertilizer reduction and organic matter addition, indicating that combined application enhances microbial nitrogen conversion capacity and reduces the risk of nitrogen loss (Wang et al., 2023). Differential expression of nitrogen cycle functional genes further reveals the soil-improving effects of applying microbial fertilizers alongside chemical fertilizers. Increased abundance of heterotrophic nitrate reductase genes (*napA*, napB etc.) and denitrification genes (*nirK/S*, *norBC*, etc.) indicates enhanced nitrogen transformation efficiency within microbial communities. While the decreased abundance of extracellular nitrate transport genes (*nrtABD*) may be related to more balanced soil nitrogen supply under combined application, reducing microbial demand for environmental nitrogen transport. This selective enrichment of functional genes reflects the targeted regulatory effect of chemical fertilizer combined with microbial fertilizer on microbial community functions.

## 5 Conclusion

This study investigated the effects of combined chemical and microbial fertilizer application on soil in intercropped peanut systems. Research findings indicate that while chemical fertilizers alone can increase readily available nutrient content, they lead to declining soil organic matter and accelerated acidification. In contrast, the combined application of chemical fertilizers and microbial fertilizer (particularly the T4 treatment: 80% chemical fertilizer + microbial fertilizer) can significantly enhance organic matter content and mitigate soil acidification trends while maintaining readily available nutrient levels in the soil. Regarding microbial communities, the T4 treatment significantly increased Shannon diversity of soil bacteria and notably elevated the abundance of the Rhizobiaceae family. Furthermore, redundancy analysis confirmed fertilizer application as a key factor in microbial community structure changes. In addition, T4 treatment significantly influenced the abundance of functional genes related to nitrogen metabolism, particularly promoting key genes involved in denitrification and nitrate reduction processes (e.g., *nirK/S*, *norBC*, and *nosZ*). These findings underscore the advantages of combined chemical and microbial fertilizer application in enhancing peanut yield and improving soil quality. These discoveries will aid in developing fertilization strategies to sustainably improve soil fertility and microbial communities, holding significant implications for promoting sustainable agricultural development.

## Data Availability

The datasets presented in this study can be found in online repositories. The names of the repository/repositories and accession number(s) can be found at: https://ngdc.cncb.ac.cn/, PRJCA051417.
